# *Streptococcus suis* Uptakes Carbohydrate Source from Host Glycoproteins by N-glycans Degradation System for Optimal Survival and Full Virulence during Infection

**DOI:** 10.3390/pathogens9050387

**Published:** 2020-05-18

**Authors:** Jiale Ma, Ze Zhang, Zihao Pan, Qiankun Bai, Xiaojun Zhong, Yinchu Zhu, Yue Zhang, Zongfu Wu, Guangjin Liu, Huochun Yao

**Affiliations:** 1 MOE Joint International Research Laboratory of Animal Health and Food Safety, College of Veterinary Medicine, Nanjing Agricultural University, Nanjing 210095, China; jialema@njau.edu.cn (J.M.); 2017107078@njau.edu.cn (Z.Z.); panzihao@njau.edu.cn (Z.P.); 2017107079@njau.edu.cn (Q.B.); 2017207027@njau.edu.cn (X.Z.); zhuyc111@163.com (Y.Z.); zhangyueruda@163.com (Y.Z.); wuzongfu@njau.edu.cn (Z.W.); liugj100@njau.edu.cn (G.L.); 2 Key Lab of Animal Bacteriology, Ministry of Agriculture, Nanjing 210095, China; 3 OIE Reference Lab for Swine Streptococcosis, Nanjing 210095, China

**Keywords:** *Streptococcus suis*, N-glycans degradation system, EndoSS, GH92, serum growth, virulence

## Abstract

Infection with the epidemic virulent strain of *Streptococcus suis* serotype 2 (SS2) can cause septicemia in swine and humans, leading to pneumonia, meningitis and even cytokine storm of Streptococcal toxic shock-like syndrome. Despite some progress concerning the contribution of bacterial adhesion, biofilm, toxicity and stress response to the SS2 systemic infection, the precise mechanism underlying bacterial survival and growth within the host bloodstream remains elusive. Here, we reported the SS2 virulent strains with a more than 20 kb *endoSS*-related insertion region that showed significantly higher proliferative ability in swine serum than low-virulent strains. Further study identified a complete N-glycans degradation system encoded within this insertion region, and found that both GH92 and EndoSS contribute to bacterial virulence, but that only DndoSS was required for optimal growth of SS2 in host serum. The supplement of hydrolyzed high-mannose-containing glycoprotein by GH92 and EndoSS could completely restore the growth deficiency of *endoSS* deletion mutant in swine serum. EndoSS only hydrolyzed a part of the model glycoprotein RNase B with high-mannose N-linked glycoforms into a low molecular weight form, and the solo activity of GH92 could not show any changes comparing with the blank control in SDS-PAGE gel. However, complete hydrolyzation was observed under the co-incubation of EndoSS and GH92, suggesting GH92 may degrade the high-mannose arms of N-glycans to generate a substrate for EndoSS. In summary, these findings provide compelling evidences that EndoSS-related N-glycans degradation system may enable SS2 to adapt to host serum-specific availability of carbon sources from glycoforms, and be required for optimal colonization and full virulence during systemic infection.

## 1. Introduction

*Streptococcus suis* is considered to be one of the important bacterial pathogens in the swine industry and also represents a significant threat to human health [1,2,3]. Many clinical manifestations caused by *S. suis* infection have been reported, such as arthritis, pneumonia, endocarditis, septicemia and meningitis [4]. Among those manifestations, septicemia is the most lethal and associated with severe economic loss [1]. Currently, host serums have been used to interact with bacterial pathogens for identification of important virulence factors [5,6,7], which contribute to developing new prevention and treatment strategies against bacterial infection. However, the underlying mechanisms of *S*. *suis* in swine serum growth and blood colonization are poorly understood and should be further explored.

To survive and even proliferate in host blood, *S*. *suis* must overcome two major obstacles, the innate immunity and nutritional limit of the host [8]. The innate immunity, such as complement-mediated opsonophagocytosis and antimicrobial peptide-mediated killing, is the first barrier of host blood that *S*. *suis* need to overcome to establish sustained bacteremia. Many of the strategies resisting the bactericidal activity of blood are in surface-exposed components, such as the bacterial capsule and cell walls anchor proteins [7,9]. Nutritional limit is the process by which nutrients are kept in various storage molecules that make them unavailable to pathogens [10]. To adapt the metal-ions limited condition, numerous metal-ions acquisition systems including *feoB*, *SSU0308* and *troA* have been reported in *S*. *suis* to uptake metal-ions from host blood during systematic infection [11,12,13]. Furthermore, nucleotide biosynthesis genes, including *purA/D*, *cdd* and *guaAB*, were reported to be critical for colonization of *S*. *suis* serotype 2 (SS2) strain S735 in septicemic mouse and pig models [14]. 

It should be noted that numerous *S. suis* genes involved in polysaccharides’ hydrolysis and the sugar phosphotransferase system were found significantly upregulated and play important roles in the septicemic process [14,15,16,17], suggesting carbohydrate metabolism may be required for the optimal survival and full virulence during bloodstream infection. The serum carbohydrate mainly contains monosaccharide, polysaccharides, glycoproteins and glycolipids [18], while only the monosaccharide such as glucose can be directly transferred and utilized by bacterial cells. Commensurate with N-glycosylation being an abundant form of protein glycosylation that also plays critical functional roles within the host including structural functions, immune response, protection of tissues, cell and molecule attachment, hormone signaling and blood coagulation [19], some host-adapted bacteria possess the mechanism to process N-linked glycans. Indeed, glycoproteins were reported to play important roles in the interaction between bacterial pathogens and hosts recently [20,21,22]. Some bacterial surface proteins have been identified to interact with host glycoproteins such as IgG, IgA or complement components for immune escape [23,24,25]. Endo-β-N-acetylglucosaminidases (ENGases) are a widely distributed class of bacterial surface hydrolases in various species, which can hydrolytically cleave β-1,4 glycosidic bonds in the inner-core region of N-glycans and release a glycan chain from their associated proteins [26]. In *Streptococcus pyogenes* and *Streptococcus dysgalactiae*, the ENGase homologs can release the fucose-containing oligosaccharides residues from IgG thus to sufficiently inhibit antibody-mediated inflammation in mouse arthritis model [25,27]. In *Streptococcus pneumoniae*, the ENGase EndoD was classified as an important component of the N-glycans processing system which targets both complex and high-mannose N-glycans from the surface of host cells for bacterial growth and full virulence during infection [28].

Here, we found the SS2 virulent strains that showed significantly higher proliferative ability in swine serum than low-virulent strains, and identified a more than 20 kb *endoSS*-related insertion region only encoded by SS2 virulent strains, which is required for the bacterial optimal proliferation in host serum. Subsequently, we explored the potential roles of this inserted *endoSS*-related cluster in SS2 virulence, bloodstream colonization during systemic infection, N-glycans degradation of model glycoproteins and host serum growth in vitro supported by host glycoproteins. Our study provides a molecular blueprint for understanding the underlying mechanisms employed by *S. suis* to survive, grow in the swine bloodstream and cause disease.

## 2. Results

### 2.1. A Significantly Higher Proliferative Ability of SS2 Virulent Strains in Swine Serum than Low-virulent Strains

**Figure 1:**
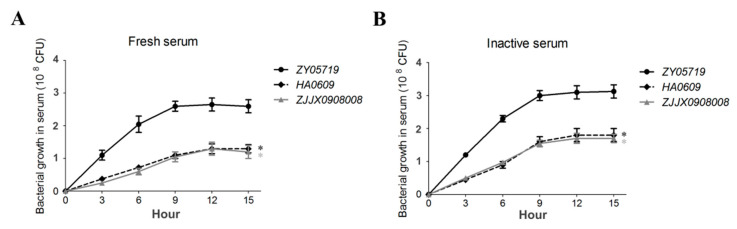
The characteristics of swine serum growth in virulent and low-virulent *S. suis* strains. Cultures of SS2 strains ZY05719, HA0609 and ZJJX0908008 were grown overnight and diluted in fresh (**A**) or inactivated (**B**) serum, and growth curves were started with ~5 × 10^5^ CFU and generated by measuring bacterial CFU/mL each three hours. Error bars represent the SDs for three independent experiments. The data were compared with that of strain ZY05719 and analyzed using the one-way ANOVA test (*, *P* < 0.05).

Our previous study revealed the genetic differences between virulent and low-virulent strains of *S. suis* serotype 2 identified by animal infection models [29]. Of these strains, ZY05719 is a high virulent one, isolated from a piglet with acute sepsis. Septicemic *S. suis* relatively adapt to the specific nutritional condition of bloodstream and are resistant to its bactericidal effect. Here, we found that this septicemic strain is capable of growing in swine serum. Two SS2 strains HA0609 and ZJJX0908008 were identified as low-virulent isolates using mouse and zebrafish infection models (Figure S1), and used for the following study. Figure 1A shows that strain ZY05719 presented a relatively better growth in whole serum in comparison with that of two low-virulent SS2 strains HA0609 and ZJJX0908008. A similar pattern is observed in inactivated serum (Figure 1B). However, this difference between strains ZY05719, HA0609 and ZJJX0908008 is not observed under the THB medium culture. 

### 2.2. The endoss Gene Encoded within a Unique Insertion Region of SS2 Virulent Strains is Required for Optimal Proliferation in Host Serum

**Figure 2:**
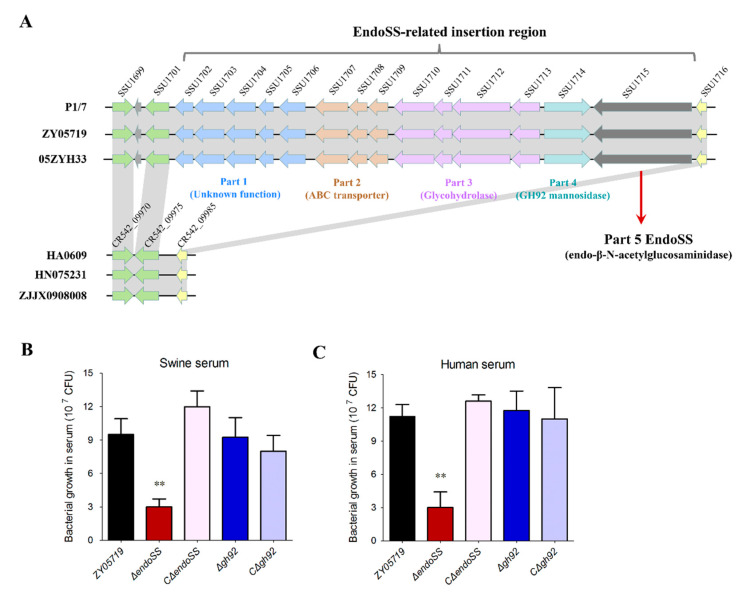
Identification of an EndoSS-related insertion region in SS2 virulent strains contributing to bacterial growth in host serum. (**A**) Schematic diagram of vicinity sequences of *endoSS* gene in virulent and low-virulent *S. suis* strains. Each colored arrow is an encoding gene, and the locus tags of genes in reference strains P1/7 and HA0609 are labeled. The regions of similar/same sequences in each zone are shaded in gray. The direction of the arrows indicates the direction of transcription. (**B**,**C**) Identification of potential serum fitness genes by deletion mutants in swine or human serum. The bacterial growth was started with ~5 × 10^5^ CFU. The growth assessment quantification was performed in host serum by measuring bacterial CFU/mL after incubation at 37 °C for 3 h. Asterisks denote significant difference in the CFU value for the mutant strains against the wild-type strain (**, *P* < 0.01). Error bars represent the SDs for three independent experiments.

A molecular epidemiological investigation of more than 100 SS2 strains identified six specific genes containing *epf*, *sly*, *rgg*, *endoSS*, *SMU_61-like* and *SpyM3_0908* that were solely encoded by virulent strains but not by low-virulent strains identified using mouse and zebrafish infection models in our previous study [29]. To further examine the genetic neighborhoods of these six genes, we found that most of the genes were located within about 2 Kb insertion regions of ZY05719 genome comparing with that of low-virulent strains HA0609, HN075231 and ZJJX0908008, only *endoSS* located within a more than 20 Kb insertion region (Figure 2A). In fact, bacterial growth deficiency always relates to nutrient limitations, and can be restored by potential salvage pathways encoded by the genes located at a similar large cluster [30]. Further analysis found that most genes within the 20 Kb *endoSS*-related insertion region are involved in polysaccharide metabolism, including regulator, glycosyl transporter and numerous glycohydrolases (Figure 2A). Two non-polar deletion mutants ∆*endoSS* and ∆*gh92* were constructed subsequently to test their growing capacity under the serum culture. As the significant deficiency on bacterial growth of low-virulent strains have been observed after incubation within swine serum at 37 °C for 3 h (Figure 1), we chose this timepoint to perform the following tests. Similar to the growth of low-virulent SS2 strains HA0609 and ZJJX0908008, the deletion of *endoSS* but not that of GH92 caused a significant growth deficiency compared with that of wild-type strain both in swine and human serum (Figure 2B,C), while its complementation completely restored this deficiency in ∆*endoSS*. However, this difference between strains wild-type and ∆*endoSS* is not observed under the THB medium culture (Figure S2), suggesting that the potential polysaccharide metabolism depending on EndoSS is only required for bacterial growth *ex vivo*.

### 2.3. EndoSS and Its Upstream GH92 Are Required for the Full Virulence of SS2 in Animal Infection Models

**Figure 3:**
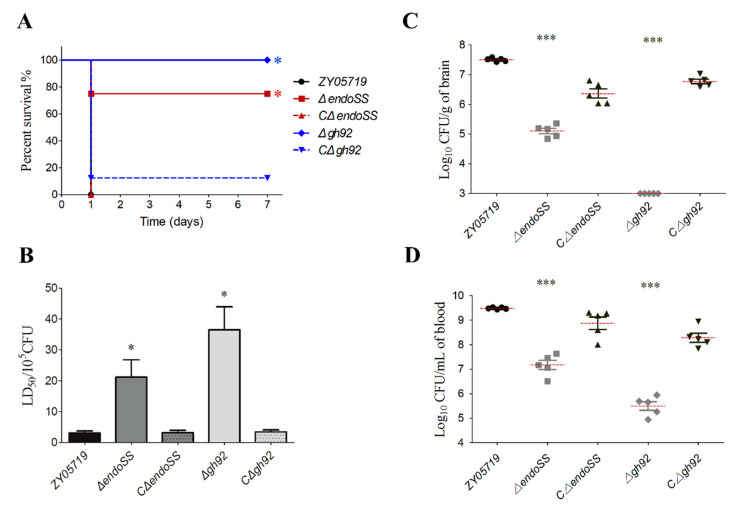
The *endoSS*-related N-glycans degradation genes were required for the full virulence in *S. suis* virulent strain ZY05719. (**A**) Survival curve of ZY05719, Δ*endoSS*, Δ*gh92* and their complemented strains in the mouse infection model. The six-week-old BALB/c mice were infected with indicated SS strains at the same dose and monitored over a 7 day period. The data were compared with that of strain ZY05719 and analyzed using the log-rank (Mantel–Cox) test (*, *P* < 0.05). (**B**) LD50 evaluation of ZY05719, Δ*endoSS*, Δ*gh92* and their complemented strains in the zebrafish infection model by monitoring over a 7-day period (*, *P* < 0.05). (**C**,**D**) Systemic infection experiments were conducted to assess bacterial proliferation in mouse blood and brain. Bacterial reisolation from the blood or brain at 12 h post-inoculation was quantified by plate count. Statistical significance was determined by a Student’s *t* test based on comparisons with the wild-type group (*** *P* < 0.001).

It should be noted that the growth defect exhibited by gene deletion in serum may manifest at the bacteremic stage of the infection, and thus, result in attenuated virulence. Thus, a mouse infection test was performed using BALB/c mice injected with 2 × 10^8^ CFU of related deletion mutant and wild-type strains. As expected, the mice infected by the Δ*endoSS* and Δ*gh92* showed a significantly higher survival rate (>70%), with or without slight clinical signs, compared with the 100% death of mice infected by the wild-type strain, which showed acute clinical signs, such as shivering, rough hair coat and depression (Figure 3A). The zebrafish is a widely recognized model for SS2 infection study [31,32]. The LD50 values for Δ*endoSS* and Δ*gh92* in zebrafish infection model were significantly increased more than 4 times compared with that of wild-type and their complemented strains (Figure 3B), which is consistent with the above results from the mouse infection model. Subsequently, we managed to further verify the role of EndoSS and GH92 in SS2 fitness in host bloodstream and organs. As shown in Figure 3C,D, the deletion of *endoSS* or *gh92* significantly attenuate the bacterial loads in mice brain and blood compared with the wild-type strain. Similar results were also observed in the spleen and kidney (Figure S3), while the above deficiencies of deletion mutants were completely restored by complementation. Altogether, our data indicated that the polysaccharide metabolism mediated by EndoSS and GH92 is important for optimal survival of SS2 in host bloodstream and organs during systemic infection.

### 2.4. Bioinformatics Analysis of an EndoSS-Related N-Glycans Degradation Gene Cluster Encoded within the Insertion Region 

**Figure 4:**
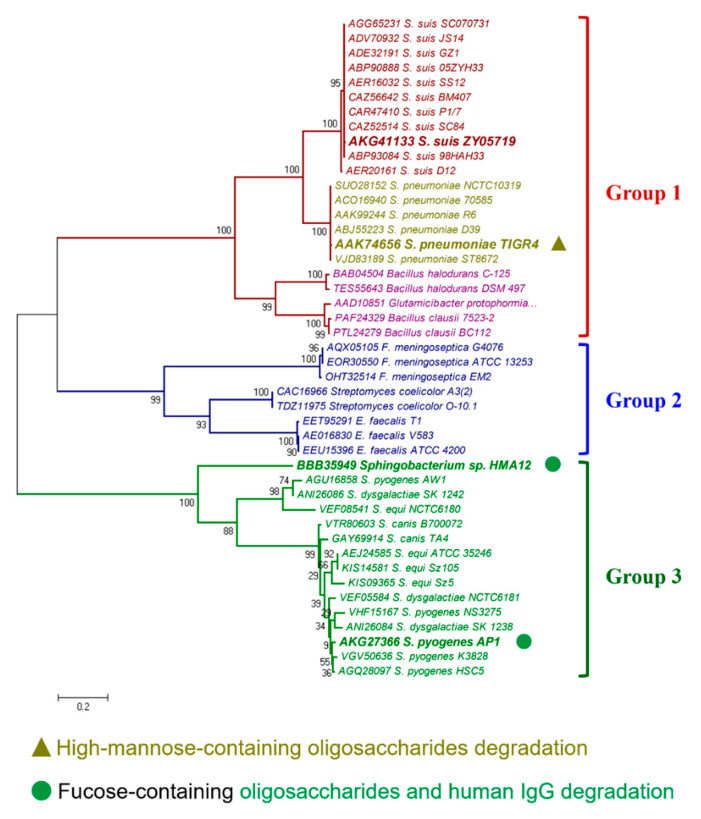
Phylogenetic analysis of EndoSS-related N-glycans degradation system. Evolutionary relationships of EndoSS homologs. A neighbor-joining tree (bootstrap n = 1000; Poisson correction) was constructed based on a ClustalW alignment of the EndoSS amino acid sequences from diverse bacterial species using the MEGA software version 5.0. The representative Endo-homologs were labeled by overstriking and increasing font size, and their potential biological activities were annotated by referring to previous reports [25,27,28,33].

**Figure 5:**
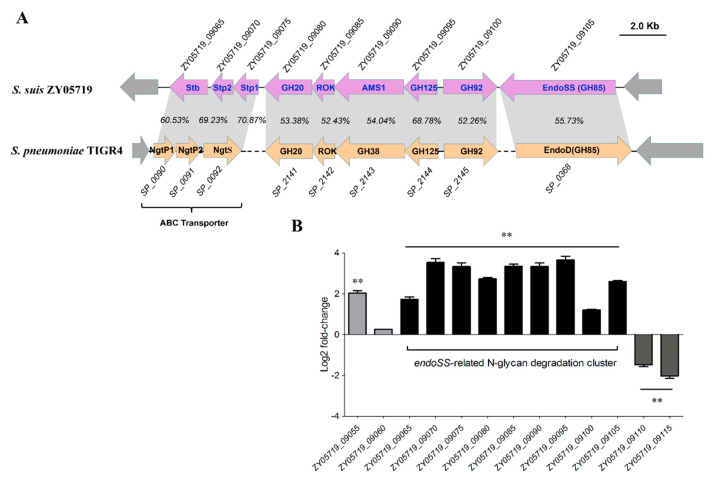
Comparative genome alignments and transcriptional activation in vivo of the *endoSS*-related N-glycans degradation gene cluster. (**A**) Schematic diagram of the genetic organization of the *endoSS*-related N-glycans degradation gene cluster in *S. suis* strain ZY05719 and *S. pneumoniae* strain TIGR4. The identity of amino acid sequences for each component of *endo*-related N-glycans degradation system are shown. (**B**) Identification of the transcriptional activation of *endoSS*-related N-glycans degradation gene cluster during infection comparing with the THB culture. The data were normalized to the housekeeping gene *parC* transcript [34]. The relative expression levels represent the mean ± SD of three biological repeats (** *P* < 0.01).

Numerous studies have reported that EndoSS-like ENGase catalyzes the hydrolysis of N-linked oligosaccharides [28,33], thereinto ENGase homologs from *S. pyogenes* and *S. dysgalactiae* can release the fucose-containing oligosaccharides residues from IgG [25,27]. Unexpectedly, both human and mouse IgG could not be catalyzed by hydrolysis of N-linked oligosaccharides by EndoSS and GH92 from *S. suis* (Figure S4). To explore why EndoSS has no IgG hydrolytic activity, a phylogenetic tree of ENGase homologs from diverse bacterial species was constructed. As shown in Figure 4, the proteins were separated into three distinct clades, namely groups 1 to 3. Notably, all EndoSS-like proteins from *S. suis* were located on the same branch (group 1) with the ENGase homologs from *S. pneumoniae* and *Bacillus species*, while exhibiting a greater evolutionary distance from the group 3 branch containing ENGase proteins from *S. pyogenes*, *S. dysgalactiae*, *S. equi*, *S. canis* and *Sphingobacterium* sp. In fact, the ENGases from *Sphingobacterium* species have been reported to only release the fucose-containing oligosaccharides residues from IgG but not the high-mannose-containing oligosaccharides residues from RNase B [33]. In *S. pneumoniae*, the EndoD homolog was classified as an important component of the N-glycans processing system which targets both complex and high-mannose N-glycans from the surface of host cells for bacterial growth and full virulence during infection [28]. To compare the genetic organization between the *S. suis* 20 Kb *endoSS*-related insertion region and the *S. pneumoniae* EndoD N-glycans processing system, their encoding genes produce the corresponding homologs including ABC transporter, GH20, ROK, GH38, GH125, GH92 and Endo-like proteins, and share high sequence identity (Figure 5A). These observations suggested that the 20 Kb insertion region of *S. suis* encodes an *endoSS*-related N-glycans degradation gene cluster. Further transcriptional analysis of this gene cluster showed that all the genes were significantly upregulated during bloodstream infection in vivo compared with the THB culture in vitro (Figure 5B), indicating it showed a potential correlation with bacterial pathogenicity.

### 2.5. EndoSS and GH92 Can Hydrolyze the N-Glycans of RNase B Collaboratively via Different Cutting Sites

**Figure 6:**
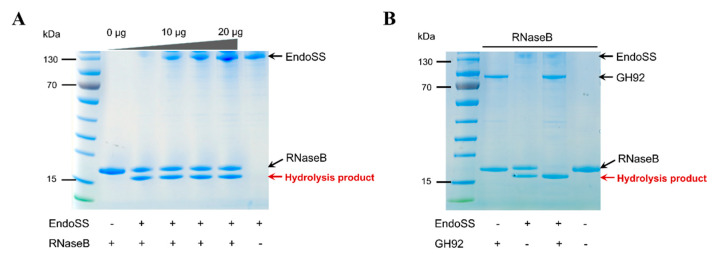
SDS-PAGE gel visualizing the glycosylated and deglycosylated RNase B following treatment with GH92 and EndoSS. (**A**) The SDS-PAGE analysis of the hydrolytic activities of recombinant EndoSS with increasing concentrations against RNase B. Native RNase B consists of Man5-Man9 glycoforms and has a mean size of approx. 18 kDa. Samples from lanes 1 to 5 showed the hydrolysis test of 6 mg/mL RNase B by adding EndoSS with 5 μg/well increment. The two bands were labeled as glycosylated and deglycosylated RNase B. (**B**) The change in size of RNase B following deglycosylation by GH92 and EndoSS is shown. Upon treatment with GH92 alone, no deglycosylated RNaseB was observed. Together, GH92 and EndoSS fully deglycosylated RNase B.

A model glycoprotein, RNase B, has a single, high-mannose N-linked glycosylation site with Man5-Man9 glycoforms [35], and have been confirmed to be hydrolyzed by *S. pneumoniae* EndoD [28]. As such, we used it as a model substrate to test the activity of GH92 and EndoSS from *S. suis* to degrade high-mannose N-glycans of glycoproteins. GH92 protein is one of the important components of EndoSS-related enzymolysis system, and predicted as an α-(1,2)-mannosidase to cleave the terminal α-(1,2)-linked mannose residues of high-mannose N-glycans. Initially, we used SDS-PAGE to observe the solo activity of EndoSS on RNase B at the concentration from 0 to 20 μg, which showed that EndoSS only hydrolyzes the RNase B partially at any concentration (Figure 6A). We then tested the combined activities of GH92 and EndoSS on RNase B, and found that the solo activity of GH92 could not produce the smaller protein band, but its product could be further hydrolyzed into a single protein band by EndoSS in the SDS-PAGE gel (Figure 6B). Indeed, the GH92 homolog from *S. pneumoniae* was confirmed to uniformly trim Man6-Man9 glycoforms down to Man5 (showing a similar band size with the untreated sample) which then fully deglycosylated by Endo-homolog to generate the single smaller band in SDS-PAGE gel [28]. These observations indicated that both GH92 and EndoSS were required for the complete conversion of RNase B into a low molecular weight form.

### 2.6. EndoSS Contributes to Optimal Growth of S. suis on a Glycoconjugate

**Figure 7:**
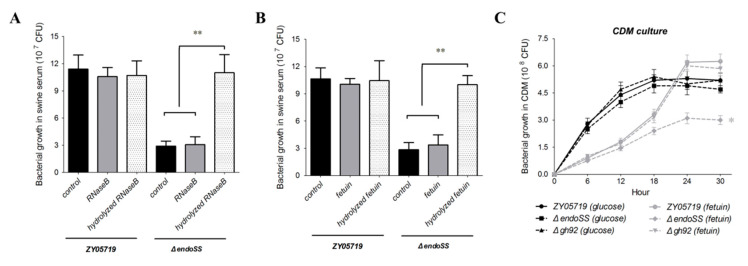
EndoSS contributes to uptakes carbohydrate source from host glycoproteins for optimal growth of *S. suis*. (**A**,**B**) The supplement of hydrolyzed RNase B and fetuin restored the growth of the *endoSS* deletion mutants in swine serum. The CFU values of the ZY05719 and Δ*endoSS* strains were measured after 3 h culture in swine serum with or without indicated supplements. Boiled reaction buffer with EndoSS and GH92 was used as control. All supplements were boiled for 5 min before using in bacterial culture. The RNase B or fetuin was hydrolyzed by EndoSS and GH92 together for 1 h. Error bars represent the SDs for three independent experiments (**, *P* < 0.01). (**C**) Growth of ZY05719 and Δ*endoSS* strains in chemically-defined medium (CDM) supplemented with the model glycoconjugate fetuin. 20 mg/mL fetuin was supplemented as the sole carbon source. The bacterial growth was started with ~5 × 10^5^ CFU. All CFU values represent the mean from three independent experiments. The data were compared with that of wild-type strain and analyzed using the one-way ANOVA test (*, *P* < 0.05).

Given that EndoSS contributes to the optimal growth of virulent SS2 in host serum, we managed to explore whether the released mannose-containing oligosaccharides residues from RNase B can restore the growth deficiency of Δ*endoSS* in this medium. Indeed, the supplementation of 6 mg/mL hydrolyzed RNase B completely recovered the growth of Δ*endoSS* to a similar level of the wild-type strain (Figure 7A), suggesting EndoSS-dependent glycosyl uptake was required to support the bacterial growth in host serum. We next asked why Δ*endoSS* showed growth deficiency in host serum but not in the THB medium (Figure 2B and Figure S2). An abundant component of host serum, fetuin, was used as a model glycoprotein for further growth tests. As shown in Figure 7B, the supplementation of 20 mg/mL hydrolyzed fetuin also completely recovered the growth of Δ*endoSS* to a similar level of the wild-type strain, suggesting the carbohydrate limit of Δ*endoSS* was overcome in serum growth. We then prepared the chemically defined medium (CDM) as previously described, and supplemented fetuin as the sole carbohydrate source to test bacterial growth in this medium, with glucose as a control here. Both Δ*endoSS* and Δ*gh92* strains were able to grow on CDM medium with fetuin, while Δ*endoSS* was unable to reach the same cell density comparing with the exhibition in wild-type and Δ*gh92* strains (Figure 7C). There was no significant difference of all strains in growth on CDM medium with glucose. All these observations suggested that several host glycoproteins with high-mannose-containing glycoforms from serum, like RNase B and fetuin, can be hydrolyzed by EndoSS related N-glycans degradation system and imported as an important carbohydrate source to support optimal survival of virulent SS2 strains during bloodstream infection.

## 3. Discussion

Bacterial survival and even proliferation in the serum is a common manifestation of a number of bacterial septicemias. Our present study demonstrated that the optimal proliferation of SS2 strain ZY05719 in swine serum in vitro or bloodstream in vivo during systemic infection relies on the EndoSS-related N-glycans degradation system. Furthermore, EndoSS and GH92 were identified to play important roles in N-glycans degradation of model glycoproteins RNase B and fetuin, which contribute to the uptake of the carbohydrate source to support bacterial growth, thus they may facilitate SS2 full virulence in mouse and zebrafish infection models.

In this study, EndoSS was confirmed that it could not hydrolyze host IgG and suppresses blood inflammation, which is not consistent with the previous studies that EndoSS-like ENGase released oligosaccharide residues from IgG, thus, to sufficiently inhibit antibody-mediated inflammation [25]. Therefore, we hypothesized that the hydrolytic activities of ENGase homologs from different bacterial species may have occurred distinct differentiation to target diverging substrates during the lengthy evolutionary process. Indeed, a recent study has reported that six Endo-homologs from *Sphingobacterium* sp., *Beauveria bassiana* and *Cordyceps militaris* only catalyze the hydrolysis of fucose-containing biantennary complex type oligosaccharides such as human IgG, but not that of high-mannose type oligosaccharides [33]. Our following phylogenetic analysis showed that all Endo-homologs were separated into three distinct clades (Figure 4), and the members with IgG hydrolytic activity from *S. pyogenes*, *S. dysgalactiae*, *S. equi*, *S. canis* and *Sphingobacterium* sp. clustered into the green branch, while the members from *S. suis*, *S. pneumoniae* and *Bacillus species* were located on the red branch. The previous studies in *S. pneumoniae* suggested the red group proteins may mainly target high-mannose N-glycans [28], while the green group proteins function for fucose-containing oligosaccharides degradation.

Numerous extracellular enzymes from diverse bacterial species involved in N-glycans degradation are at least to some degree associated with the bacterial virulence [36,37]. Our results showed a significant upregulation of the whole *endoSS-*related N-glycans degradation gene cluster during bloodstream infection in vivo compared with the THB culture in vitro (Figure 5B), and further confirmed this system to closely relate with bacterial pathogenicity in animal infection models. Unexpectedly, the Δ*gh92* was particularly striking in its greater effect than Δ*endoSS* to reduce the virulence both in mouse survival curve and zebrafish LD50 evaluation. In *S. pneumoniae*, the homolog of GH92 has been identified to trim the terminal α-(1,2)-linked mannose residues of high-mannose N-glycans to generate Man_5_GlcNAc_2_, which is a substrate for EndoD homolog [28]. The GH92 proteins from *S. suis* shared the highest sequence identity with the homolog from *S. pneumoniae*. Therefore, the glycoforms of RNase B may be uniformly trimmed down to Man5 after treatment with GH92 of SS2, then could be fully deglycosylated by EndoSS to generate the single smaller band in the SDS-PAGE gel. However, EndoSS is only able to cleave the chitobiose core of the Man5 glycoforms, which causes the residual Man6-Man9 glycoforms to remain intact, thus, two bands for RNase B (glycosylated and deglycosylated) are observed after treatment, which also is consistent with the previous report of *S. pneumoniae* [28]. More types of high-mannose-containing glycoforms (≥Man6) can be trimmed by GH92 than EndoSS, which may extremely destroy the biological functions of target glycoproteins or glycolipids, thus, most profoundly facilitate bacterial virulence. Indeed, the pathogenic phenotypes rendered by the deletion of *endoSS* or *gh92* result from the effects of altering the structure of glycans in the host, rather than carbon source uptake or energy liberation, and the cleavage of terminal α-1,2-mannose linkages is extremely important for the bacterial interaction with its host in an animal infection model. Nevertheless, the relevant in vivo glycoconjugate targets of EndoSS and GH92, indeed, all of the enzymatic components of the N-glycans degradation system of *S. suis* or other bacterial pathogens, remain to be further explored.

In this study, we mainly managed to clarify the roles of EndoSS-related N-glycans degradation system played in supporting bacterial serum growth. The above descriptions indicate that GH92 is able to degrade the high-mannose arms of N-glycans to generate a substrate for EndoSS, suggesting the functional association between these two enzymes. EndoSS possesses a cell-wall anchoring LPXTG motif and a YSIRK secretion signal peptide, thus, it is considered as a part of the extracellular landscape of the bacterium. GH92 should also be an extracellular enzyme so that it can function upstream of EndoSS in the N-glycans degradation system. Unexpectedly, we could not find any known secretion signals in the N-terminal sequence of GH92, and predicted its subcellular localization both in cytoplasmic and extracellular spaces using PSORTb v.2.0 [38] and SubLoc [39] software. However, ZY05719_09080/GH20, ZY05719_09090/GH38 and ZY05719_09095/GH125 were predicted as cytoplasmic proteins using the same method, which is consistent with their function located at the downstream of EndoSS. In fact, the capacity using an unknown mechanism to non-classically secrete some proteins is well documented in *Streptococcus* species [40,41,42], and GH92 may be one of these proteins. Another concerned result is that only Δ*endoSS* but not Δ*gh92*, showed significant growth deficiency in host serum and the CDM medium with fetuin as the sole carbon source. A potential reason is that the solo activity of EndoSS still can release polysaccharide residues from serum glycans or supplemented fetuin to satisfy bacterial growth needs of carbon source. Indeed, host serum may contain abundant glycan targets of EndoSS, such as complement component C3, that have been reported to be decorated with mannose-containing N-glycans [43], which may be cleaved to release enough source.

The putative ABC transporter of Man_3/5_GlcNAc encoded by ZY05719_09065/70/75 is the important component of EndoSS-related N-glycans degradation system in this study. In *S. pneumoniae*, the inactivation of ZY05719_09065/70/75 homolog was confirmed to reduce growth in the CDM medium with fetuin as sole carbon source, while was unnecessary for bacterial full virulence [28]. Unexpectedly, we could not construct the *zy05719_09065/70/75* deletion mutant for following functional verification in *S. suis*. Through at least 15 attempts, no deletion clones could be obtained either by the way of suicide vector pSET-4s or ComRS natural transformation [44,45], suggesting this ABC transporter may be essential for survival in SS2 strains. The deletion of *zy05719_09065/70/75* may cause the destruction of structural integrity of the cytomembrane or cell wall, which partially explained our failure in genetic deletion. Otherwise, it is speculated that some important carbon sources for bacterial growth may be the substrates of this ABC transporter, which are not just limited to transport the Man_3/5_GlcNAc. 

In summary, our study identified an N-glycans degradation system within a more than 20 kb *endoSS*-related insertion region only encoded by SS2 virulent strains, and comprehensively examined its role in trimming high-mannose-containing glycoforms, bacterial serum growth and pathogenicity. These findings provide compelling evidence that an EndoSS-related N-glycans degradation system may enable SS2 to adapt to host serum-specific availability of carbon sources, and be required for optimal colonization and full virulence during systemic infection. These related factors may serve as therapeutic targets for countering or preventing SS2 serum fitness in the clinic.

## 4. Materials and Methods 

### 4.1. Bacterial Strains, Plasmids and Growth Conditions

Bacterial strains and plasmids used in this study are listed in Table S1. For genetic manipulations, all strains were grown on LB medium at 37 °C with aeration, supplemented with kanamycin (Kan, 50 µg/mL), ampicillin (Amp, 100 µg/mL), chloramphenicol (Clm, 25 µg/mL), nalidixic acid (Nal, 50 µg/mL) or 0.1 mM isopropyl–D-thiogalactopyranoside (IPTG) when necessary.

### 4.2. Swine Blood, Serum and Animals

Swine blood was collected to prepare serum from healthy pigs that tested negative for SS serotype 2, as determined by ELISA [17,46]. Six-week-old female specific pathogen free (SPF) BALB/c mice were purchased from Yangzhou University (Comparative Medicine Center). Adult healthy zebrafish were obtained from Nanjing EzeRinka Biotechnology Co Ltd. All animal experiments were approved by the Ethics Committee for Animal Experimentation of Nanjing Agricultural University and conducted in strict accordance with the animal welfare standards of the Animal Research Committee Guidelines of Jiangsu Province (License Number: SYXK (SU) 2017-0007).

### 4.3. DNA Manipulations and Plasmids Construction

DNA amplification, ligation and electroporation were performed as previously described [47] unless otherwise indicated. All restriction and DNA-modifying enzymes were purchased from TaKaRa and performed according to supplier instructions. Deletion mutants were constructed using the natural transformation method, according to two recent studies [45,48]. The forward and reverse homologous sequences of the target gene were fused with the chloramphenicol marker by overlap PCR. Then, the DNA products were mixed with the peptide and wild-type bacterial cells, incubated, and selected on THB agar (Cm^R^). For complementation, the PCR fragments of target genes (including putative promoter sequences) were cloned into the pSET2 vector. After transformation into *E. coli* Top10 for propagation, the recombinant plasmid was electroporated into mutant competent cells. DNA sequencing was performed by Genscript Biotechnology Co Ltd (Nanjing, China).

### 4.4. Bioinformatics Identification of EndoSS-Related N-Glycans Degradation System

Protein sequences of *Streptococcus* N-glycans degradation system were retrieved from the National Center for Biotechnology Information (NCBI) database, and the corresponding locus_tags are listed in figures. Their functional prediction was performed using HHpred and Phyre2 [49,50]. Using these screened conserved proteins from the *Streptococcus* N-glycans degradation system, BLASTP analyses were performed against the non-redundant protein database (ftp://ftp.ncbi.nih.gov/blast/db/) to identify their homologs. Phylogenetic analyses were performed following the procedures outlined by Bingle et al. [51]. A ClustalW alignment was generated using the EndoSS or GH92 amino-acid sequences. A phylogenetic tree was constructed using MEGA 7.0 with the neighbor-joining method with Poisson correction and 1000 bootstrap replicates.

### 4.5. RNA Isolation and qRT-PCR Analysis

Total RNA was extracted using TRIzol reagent (Vazyme, China) according to the manufacturer’s instructions, and residual genomic DNA was then removed by digestion with DNase I (TaKaRa). cDNA was synthesized using the HiScriptII first-strand cDNA synthesis kit (Vazyme). The relative amount of target gene mRNA was normalized to the housekeeping gene *parC* transcript [34]. The relative fold change was calculated by the threshold cycle (2^−ΔΔCT^) method [52]. The reported values represented the mean ± SD of three independent RNA extractions.

### 4.6. Bacterial Culture in Swine Serum in vitro

Bacterial strains were cultured to the exponential growth phase (OD600nm~0.6), pelleted by centrifugation at 8000 rpm for 5 min, washed twice in PBS, and then resuspended in an equal volume (5 mL) of freshly isolated swine serum. Mixtures were incubated at 37 °C with occasional gentle shaking to avoid sedimentation. An aliquot of the infected swine serum culture was taken out at the designed time points, and the number of viable bacteria was determined by plating serial dilutions onto THB plates and incubating overnight at 37 °C. 

### 4.7. S. suis Growth Assays in Specific Mediums with Indicated Glycoproteins

Chemically defined media (CDM) was prepared as previously described [53], at 2.5 × concentration to allow addition of sufficient carbohydrate to support bacterial growth. Swine serum also was used as a specific medium after supplement of indicted glycoproteins for further test of growth characteristics. The medium with or without recombinant EndoSS was supplemented with glucose (12 mM), RNase B (6 mg mL^−1^, Sigma) or fetuin (20 mg mL^−1^, Sigma). *S. suis* strains were grown in THB to the exponential growth phase (OD600nm~0.6), and washed and resuspended by PBS, then inoculated into 1 mL CDM or swine serum supplemented with the appropriate carbon source at the ratio of 1:100. The supplementation of glucose within the medium served as a positive control, demonstrating in each experiment that mutant strains showed no general growth defect relative to the wild-type strain. Finally, the number of viable bacteria was determined by plating serial dilutions onto THB plates and incubating overnight at 37 °C. Medium with no added carbohydrate served as a negative control.

### 4.8. Preparation of Recombinant EndoSS and GH92 Glycosyl Hydrolases

The construction of recombinant plasmids (primers in Table S2) and protein expression were performed following standard molecular cloning procedures. The protein was purified under non-denaturing conditions by Ni-NTA Spin Columns (QIAGEN) from BL21 Star (DE3) carrying the recombinant pCold-II plasmid after IPTG induction (0.1mM) for 20h at 16 °C, and then ultrafiltered using 30.0-kD cutoff spin columns (Millipore) to maintain homogeneity. Obtained proteins were quantified by absorbance at 280 nm for further hydrolysis experiments.

### 4.9. Analysis of Hydrolytic Activity of EndoSS and GH92 Enzymes

To determine whether the recombinant EndoSS and GH92 proteins could hydrolyze glycoproteins, each recombinant protein was mixed with 0–20μg of RNase B (Sigma) at a final concentration of 25 mM in 50μL of reaction buffer (pH 7.4), and incubated for 3 h at 37 °C. Moreover, the hydrolysis experiments of human and mouse IgG (NEB) were performed by mixing with these recombinant hydrolases similarly. Subsequently, all reaction samples were subjected to SDS-PAGE, following which gels were stained with Coomassie Brilliant Blue (CBB) EzStain AQua (Atto).

### 4.10. Animal Infection Assays

Zebrafish and BABL/c mice infection assays were carried out as described previously [54]. Ten mice in each group were challenged by intraperitoneal injection with the indicted strain at a dose of 5 × 10^8^ CFU/mouse and monitored for symptoms until seven days postinfection. The negative control group was challenged with an equal volume of sterile PBS. Zebrafish were divided into four groups and were inoculated with 1.0 × 10^7^, 1.0 × 10^6^ or 1.0 × 10^5^ CFU/fish or PBS (control) by intraperitoneal injection (0.02 mL/fish). Fifteen fish were used per dose, and the number of deaths was recorded to calculate half the lethal dose until seven days postinfection. To evaluate bacterial proliferation in vivo, the bacterial load assay was conducted. Five mice in each group were inoculated with 3 × 10^8^ CFU of indicted strain, blood and related organs were harvested, weighed, and homogenized in PBS at 6, 12 and 18 h postinfection, respectively. After that, the homogenized samples were serially diluted and plated on THA to enumerate the CFU.

### 4.11. Statistical Analysis

Statistical analyses were performed using Prism 5.0 (GraphPad, LaJolla, CA, USA). Two-way ANOVA was performed for the qRT-PCR results, and one-way ANOVA was used for the bacterial survival assay in fresh swine serum under static culture. Data from in vivo colonization assays were analyzed by Mann–Whitney two-tailed U tests. Differences were defined as significant at *P* < 0.05 and indicated by ‘*’ or ‘**’.

## Supplementary Materials

The following are available online at https://www.mdpi.com/2076-0817/9/5/387/s1, Table S1: Summary of bacterial strains and plasmids for this study, Table S2: Primers used for PCR amplification, Figure S1: Identification of two low-virulent SS2 isolates using mouse and zebrafish infection models, Figure S2: Growth curve of wild-type, deletion mutant and complemented strains, Figure S3: Survival of wild-type, deletion mutant and complemented strains in mice organs, Figure S4: SDS-PAGE analysis of the hydrolytic activities of recombinant proteins against human IgG, and RT-qPCR analysis of Inflammatory factors’ transcription.

## References

[1] Goyette-Desjardins G., Auger J.P., Xu J., Segura M., Gottschalk M. (2014). *Streptococcus suis*, an important pig pathogen and emerging zoonotic agent-an update on the worldwide distribution based on serotyping and sequence typing. Emerg. Microbes Infect..

[2] Segura M. (2009). *Streptococcus suis*: An emerging human threat. J. Infect. Dis..

[3] Wertheim H.F., Nghia H.D., Taylor W., Schultsz C. (2009). *Streptococcus suis*: An emerging human pathogen. Clin. Infect. Dis..

[4] Feng Y., Zhang H., Wu Z., Wang S., Cao M., Hu D., Wang C. (2014). *Streptococcus suis* infection: An emerging/reemerging challenge of bacterial infectious diseases?. Virulence.

[5] Smith H.E., Buijs H., de Vries R., Wisselink H.J., Stockhofe-Zurwieden N., Smits M.A. (2001). Environmentally regulated genes of *Streptococcus suis*: Identification by the use of iron-restricted conditions in vitro and by experimental infection of piglets. Microbiology.

[6] Ma J., An C., Jiang F., Yao H., Logue C., Nolan L.K., Li G. (2018). Extraintestinal pathogenic Escherichia coli increase extracytoplasmic polysaccharide biosynthesis for serum resistance in response to bloodstream signals. Mol. Microbiol..

[7] Phan M.D., Peters K.M., Sarkar S., Lukowski S.W., Allsopp L.P., Gomes Moriel D., Achard M.E., Totsika M., Marshall V.M., Upton M. (2013). The serum resistome of a globally disseminated multidrug resistant uropathogenic Escherichia coli clone. PLoS Genet..

[8] Huja S., Oren Y., Biran D., Meyer S., Dobrindt U., Bernhard J., Becher D., Hecker M., Sorek R., Ron E.Z. (2014). Fur is the master regulator of the extraintestinal pathogenic Escherichia coli response to serum. mBio.

[9] Gu H., Zhu H., Lu C. (2009). Use of in vivo-induced antigen technology (IVIAT) for the identification of *Streptococcus suis* serotype 2 in vivo-induced bacterial protein antigens. BMC Microbiol..

[10] Weinberg E.D. (2009). Iron availability and infection. Biochim. Et Biophys. Acta.

[11] Aranda J., Cortes P., Garrido M.E., Fittipaldi N., Llagostera M., Gottschalk M., Barbe J. (2009). Contribution of the FeoB transporter to *Streptococcus suis* virulence. Int. Microbiol..

[12] Wichgers Schreur P.J., Rebel J.M., Smits M.A., van Putten J.P., Smith H.E. (2011). TroA of *Streptococcus suis* is required for manganese acquisition and full virulence. J. Bacteriol..

[13] Aranda J., Teixido L., Fittipaldi N., Cortes P., Llagostera M., Gottschalk M., Barbe J. (2012). Inactivation of the gene encoding zinc-binding lipoprotein 103 impairs the infectivity of *Streptococcus suis*. Can. J. Vet. Res. Rev. Can. De Rech. Vet..

[14] Wilson T.L., Jeffers J., Rapp-Gabrielson V.J., Martin S., Klein L.K., Lowery D.E., Fuller T.E. (2007). A novel signature-tagged mutagenesis system for *Streptococcus suis* serotype 2. Vet. Microbiol..

[15] Deutscher J., Ake F.M., Derkaoui M., Zebre A.C., Cao T.N., Bouraoui H., Kentache T., Mokhtari A., Milohanic E., Joyet P. (2014). The bacterial phosphoenolpyruvate:carbohydrate phosphotransferase system: Regulation by protein phosphorylation and phosphorylation-dependent protein-protein interactions. Microbiol. Mol. Biol. Rev. MMBR.

[16] Li W., Liu L., Qiu D., Chen H., Zhou R. (2010). Identification of *Streptococcus suis* serotype 2 genes preferentially expressed in the natural host. Int. J. Med Microbiol..

[17] Wu Z., Wu C., Shao J., Zhu Z., Wang W., Zhang W., Tang M., Pei N., Fan H., Li J. (2014). The *Streptococcus suis* transcriptional landscape reveals adaptation mechanisms in pig blood and cerebrospinal fluid. RNA.

[18] Reitsma S., Slaaf D.W., Vink H., van Zandvoort M.A., oude Egbrink M.G. (2007). The endothelial glycocalyx: Composition, functions, and visualization. Pflug. Arch. Eur. J. Physiol..

[19] Helenius A., Aebi M. (2001). Intracellular functions of N-linked glycans. Science.

[20] Manfredi P., Renzi F., Mally M., Sauteur L., Schmaler M., Moes S., Jeno P., Cornelis G.R. (2011). The genome and surface proteome of Capnocytophaga canimorsus reveal a key role of glycan foraging systems in host glycoproteins deglycosylation. Mol. Microbiol..

[21] Jimenez-Munguia I., Pulzova L., Kanova E., Tomeckova Z., Majerova P., Bhide K., Comor L., Sirochmanova I., Kovac A., Bhide M. (2018). Proteomic and bioinformatic pipeline to screen the ligands of S. pneumoniae interacting with human brain microvascular endothelial cells. Sci. Rep..

[22] Cao Y., Rocha E.R., Smith C.J. (2014). Efficient utilization of complex N-linked glycans is a selective advantage for Bacteroides fragilis in extraintestinal infections. Proc. Natl. Acad. Sci. USA.

[23] Lynskey N.N., Reglinski M., Calay D., Siggins M.K., Sriskandan S. (2017). Multi-functional mechanisms of immune evasion by the streptococcal complement inhibitor C5a peptidase. PLoS Pathog..

[24] Fu L., Zhao J., Lin L., Zhang Q., Xu Z., Han L., Xie C., Zhou R., Jin M., Zhang A. (2015). Characterization of IgA1 protease as a surface protective antigen of *Streptococcus suis* serotype 2. Microbes Infect..

[25] Nandakumar K.S., Collin M., Happonen K.E., Lundstrom S.L., Croxford A.M., Xu B., Zubarev R.A., Rowley M.J., Blom A.M., Kjellman C. (2018). Streptococcal Endo-beta-N-Acetylglucosaminidase Suppresses Antibody-Mediated Inflammation In Vivo. Front. Immunol..

[26] Fairbanks A.J. (2017). The ENGases: Versatile biocatalysts for the production of homogeneous N-linked glycopeptides and glycoproteins. Chem. Soc. Rev..

[27] Shadnezhad A., Naegeli A., Sjogren J., Adamczyk B., Leo F., Allhorn M., Karlsson N.G., Jensen A., Collin M. (2016). EndoSd: An IgG glycan hydrolyzing enzyme in Streptococcus dysgalactiae subspecies dysgalactiae. Future Microbiol..

[28] Robb M., Hobbs J.K., Woodiga S.A., Shapiro-Ward S., Boraston A.B. (2017). Molecular Characterization of N-glycan Degradation and Transport in *Streptococcus pneumoniae* and Its Contribution to Virulence. PLoS Pathog..

[29] Dong W., Ma J., Zhu Y., Zhu J., Yuan L., Wang Y., Xu J., Pan Z., Wu Z., Zhang W. (2015). Virulence genotyping and population analysis of *Streptococcus suis* serotype 2 isolates from China. Infect. Genet. Evol. J. Mol. Epidemiol. Evol. Genet. Infect. Dis..

[30] Cai W., Wannemuehler Y., Dell’anna G., Nicholson B., Barbieri N.L., Kariyawasam S., Feng Y., Logue C.M., Nolan L.K., Li G. (2013). A novel two-component signaling system facilitates uropathogenic Escherichia coli’s ability to exploit abundant host metabolites. PLoS Pathog..

[31] Neely M.N., Pfeifer J.D., Caparon M. (2002). Streptococcus-zebrafish model of bacterial pathogenesis. Infect. Immun..

[32] Wu Z., Zhang W., Lu Y., Lu C. (2010). Transcriptome profiling of zebrafish infected with *Streptococcus suis*. Microb. Pathog..

[33] Huang Y., Higuchi Y., Kinoshita T., Mitani A., Eshima Y., Takegawa K. (2018). Characterization of novel endo-beta-N-acetylglucosaminidases from Sphingobacterium species, Beauveria bassiana and Cordyceps militaris that specifically hydrolyze fucose-containing oligosaccharides and human IgG. Sci. Rep..

[34] Zhong X., Zhang Y., Zhu Y., Dong W., Ma J., Pan Z., Yao H. (2019). Identification of an Autorepressing Two-Component Signaling System That Modulates Virulence in *Streptococcus suis* Serotype 2. Infect. Immun..

[35] Rudd P.M., Scragg I.G., Coghill E., Dwek R.A. (1992). Separation and analysis of the glycoform populations of ribonuclease B using capillary electrophoresis. Glycoconj. J..

[36] Dalia A.B., Standish A.J., Weiser J.N. (2010). Three surface exoglycosidases from *Streptococcus pneumoniae*, NanA, BgaA, and StrH, promote resistance to opsonophagocytic killing by human neutrophils. Infect. Immun..

[37] Obert C., Sublett J., Kaushal D., Hinojosa E., Barton T., Tuomanen E.I., Orihuela C.J. (2006). Identification of a Candidate *Streptococcus pneumoniae* core genome and regions of diversity correlated with invasive pneumococcal disease. Infect. Immun..

[38] Gardy J.L., Laird M.R., Chen F., Rey S., Walsh C.J., Ester M., Brinkman F.S. (2005). PSORTb v.2.0: Expanded prediction of bacterial protein subcellular localization and insights gained from comparative proteome analysis. Bioinformatics.

[39] Chen H., Huang N., Sun Z. (2006). SubLoc: A server/client suite for protein subcellular location based on SOAP. Bioinformatics.

[40] Jeong J.K., Kwon O., Lee Y.M., Oh D.B., Lee J.M., Kim S., Kim E.H., Le T.N., Rhee D.K., Kang H.A. (2009). Characterization of the *Streptococcus pneumoniae* BgaC protein as a novel surface beta-galactosidase with specific hydrolysis activity for the Galbeta1-3GlcNAc moiety of oligosaccharides. J. Bacteriol..

[41] Perez-Dorado I., Galan-Bartual S., Hermoso J.A. (2012). Pneumococcal surface proteins: When the whole is greater than the sum of its parts. Mol. Oral Microbiol..

[42] Holmes A.R., McNab R., Millsap K.W., Rohde M., Hammerschmidt S., Mawdsley J.L., Jenkinson H.F. (2001). The pavA gene of *Streptococcus pneumoniae* encodes a fibronectin-binding protein that is essential for virulence. Mol. Microbiol..

[43] Hirani S., Lambris J.D., Muller-Eberhard H.J. (1986). Structural analysis of the asparagine-linked oligosaccharides of human complement component C3. Biochem. J..

[44] Takamatsu D., Osaki M., Sekizaki T. (2001). Thermosensitive suicide vectors for gene replacement in *Streptococcus suis*. Plasmid.

[45] Zhu Y., Dong W., Ma J., Zhang Y., Pan Z., Yao H. (2019). Utilization of the ComRS system for the rapid markerless deletion of chromosomal genes in *Streptococcus suis*. Future Microbiol..

[46] Wei Z., Cheng P.L. (2007). Immunoproteomics of extracellular proteins of Chinese virulent strains of *Streptococcus suis* type 2. Proteomics.

[47] Ma J., Bao Y., Sun M., Dong W., Pan Z., Zhang W., Lu C., Yao H. (2015). Two Functional Type VI Secretion Systems in Avian Pathogenic Escherichia coli Are Involved in Different Pathogenic Pathways. Infect. Immun..

[48] Zaccaria E., Wels M., van Baarlen P., Wells J.M. (2016). Temporal Regulation of the Transformasome and Competence Development in *Streptococcus suis*. Front. Microbiol..

[49] Kelley L.A., Mezulis S., Yates C.M., Wass M.N., Sternberg M.J.E. (2015). The Phyre2 web portal for protein modeling, prediction and analysis. Nat. Protoc..

[50] Kelley L.A., Sternberg M.J.E. (2009). Protein structure prediction on the Web: A case study using the Phyre server. Nat. Protoc..

[51] Bingle L.E., Bailey C.M., Pallen M.J. (2008). Type VI secretion: A beginner’s guide. Curr. Opin. Microbiol..

[52] Livak K., Schmittgen T. (2000). Analysis of Relative Gene Expression Data Using Real-Time Quantitative PCR and the 2^−^^ΔΔCt^ Method. Methods.

[53] Kloosterman G.T. (2006). To have neighbour’s fare: Extending the molecular toolbox for *Streptococcus pneumoniae*. Microbiology.

[54] Zhu Y., Zhang Y., Ma J., Dong W., Zhong X., Pan Z., Yao H. (2019). ICESsuHN105, a Novel Multiple Antibiotic Resistant ICE in *Streptococcus suis* Serotype 5 Strain HN105. Front. Microbiol..

