# Supplementary figures and images for: Streptococcus suis Uptakes Carbohydrate Source from Host Glycoproteins by N-glycans Degradation System for Optimal Survival and Full Virulence during Infection
Source: Pathogens. 2020 May 18;9(5):387. doi: 10.3390/pathogens9050387 (PMC7281376; doi:10.3390/pathogens9050387)

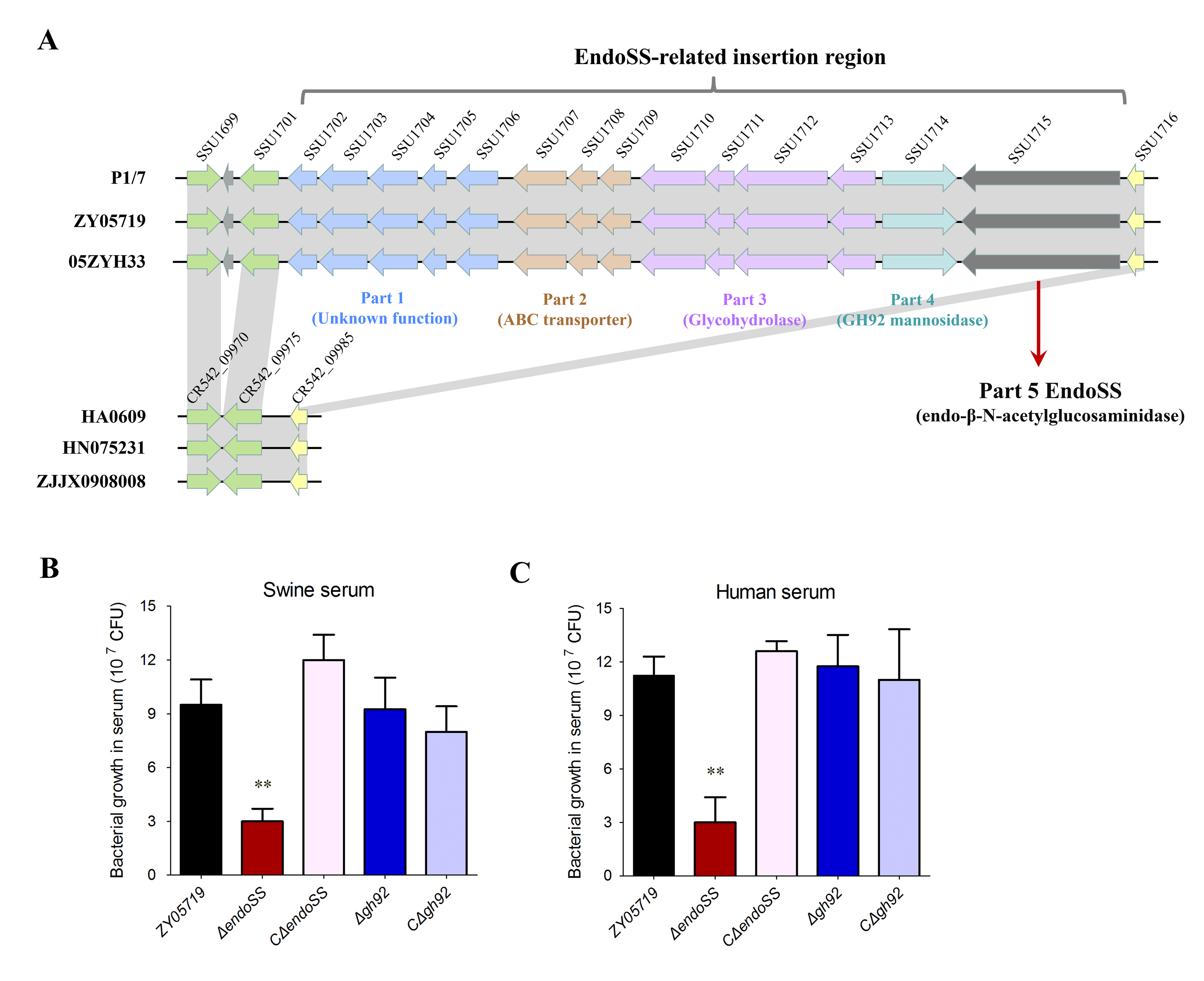

Supplement: Supplementary file 1 [file pathogens-09-00387-s001.zip › Figures/Fig. 2.tif]

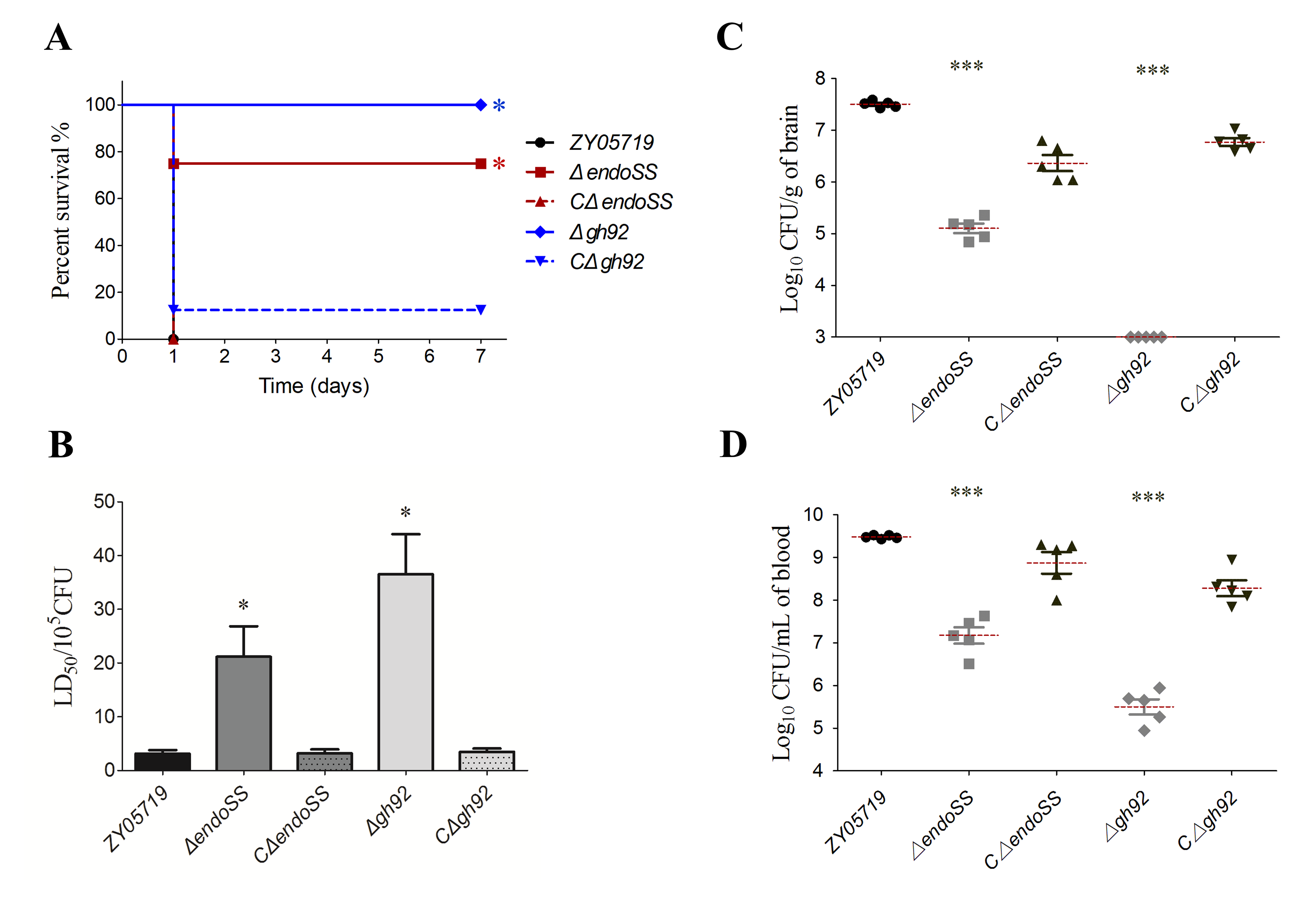

Supplement: Supplementary file 1 [file pathogens-09-00387-s001.zip › Figures/Fig. 3.tif]

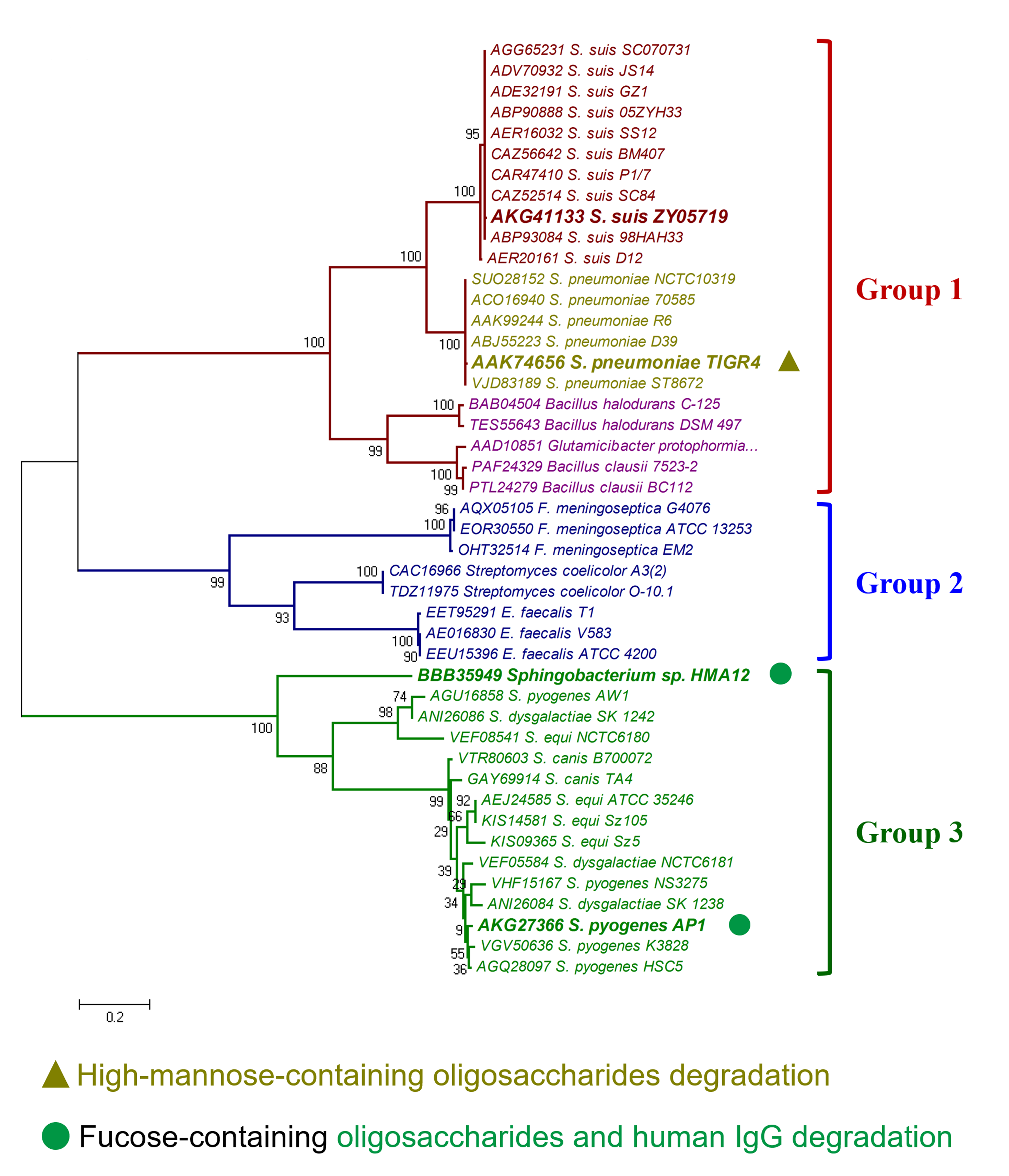

Supplement: Supplementary file 1 [file pathogens-09-00387-s001.zip › Figures/Fig. 4.tif]

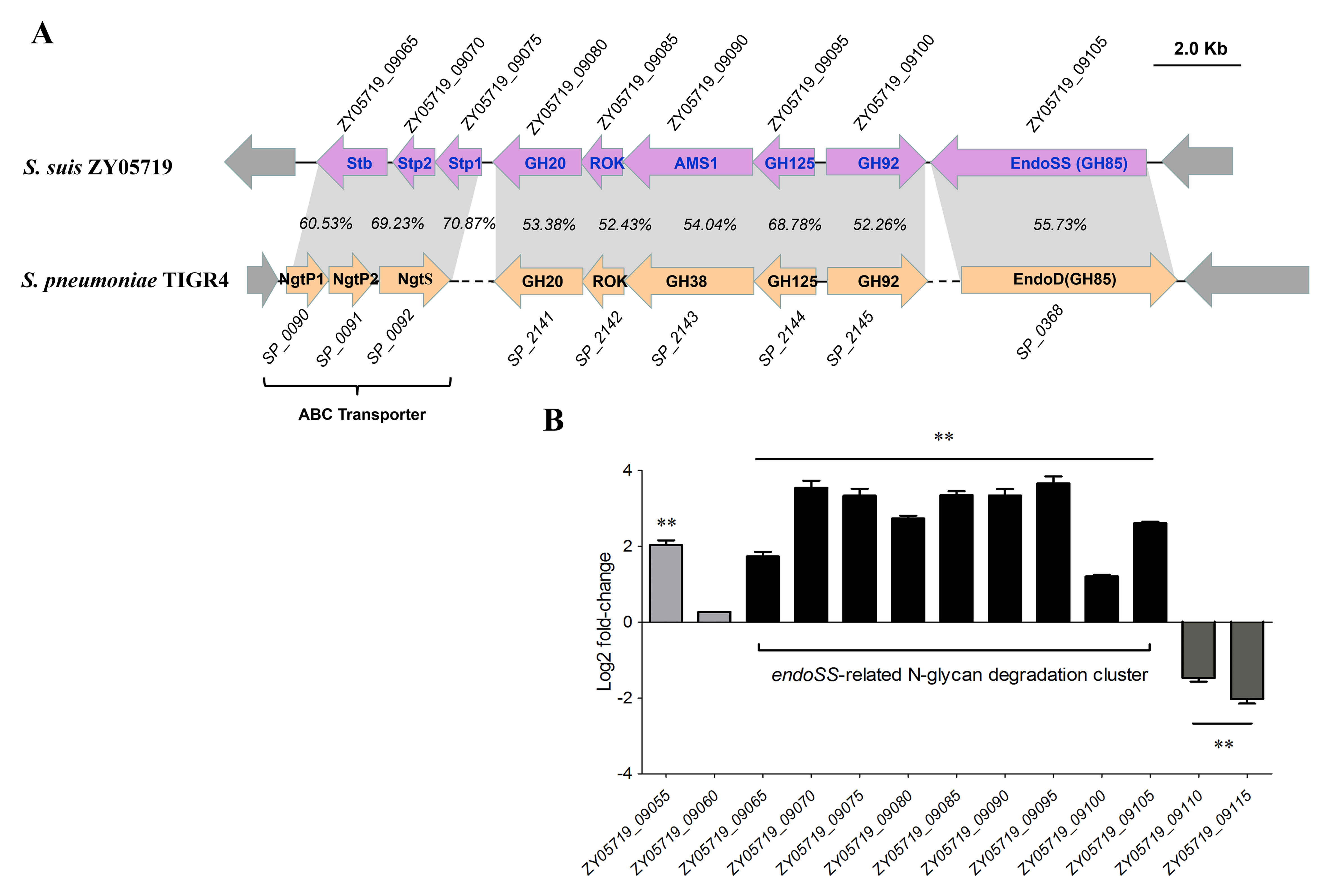

Supplement: Supplementary file 1 [file pathogens-09-00387-s001.zip › Figures/Fig. 5.tif]

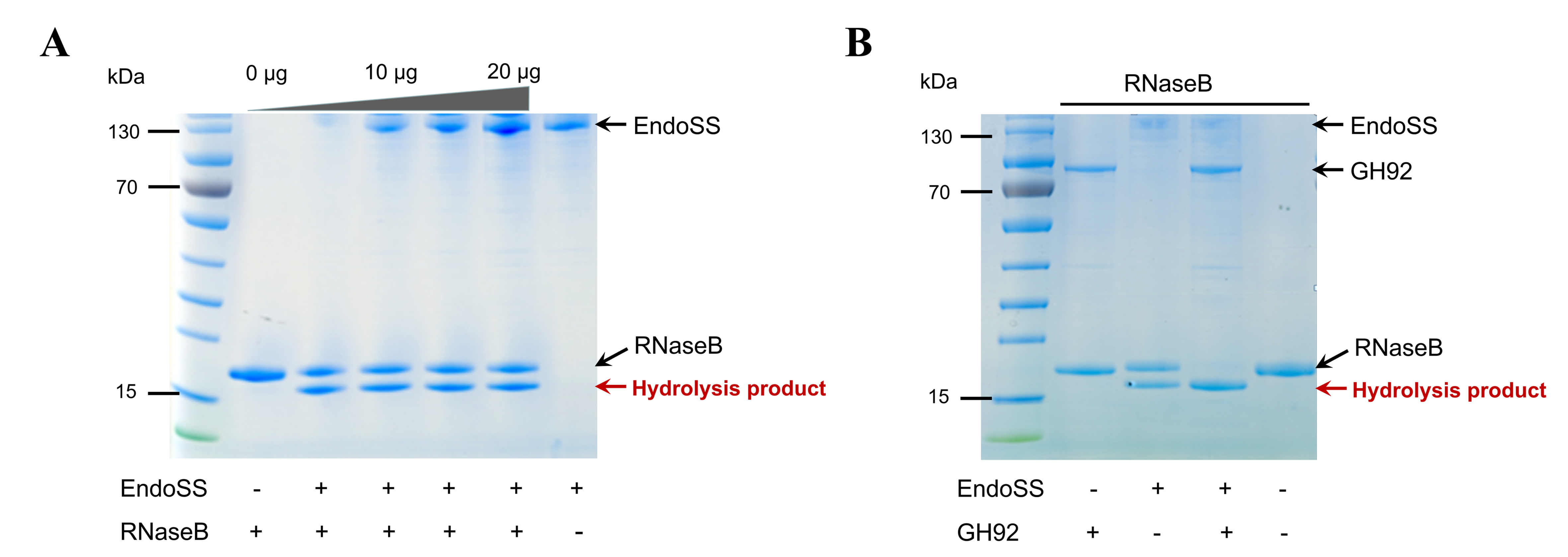

Supplement: Supplementary file 1 [file pathogens-09-00387-s001.zip › Figures/Fig. 6.tif]

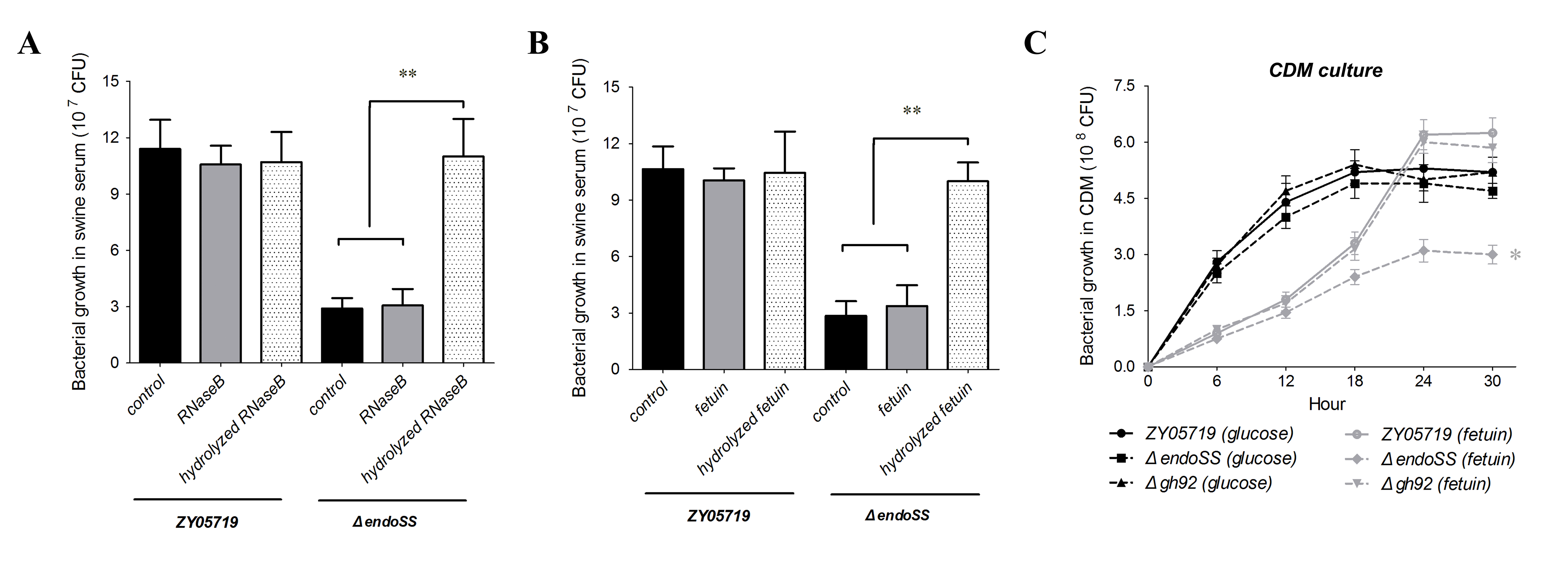

Supplement: Supplementary file 1 [file pathogens-09-00387-s001.zip › Figures/Fig. 7.tif]

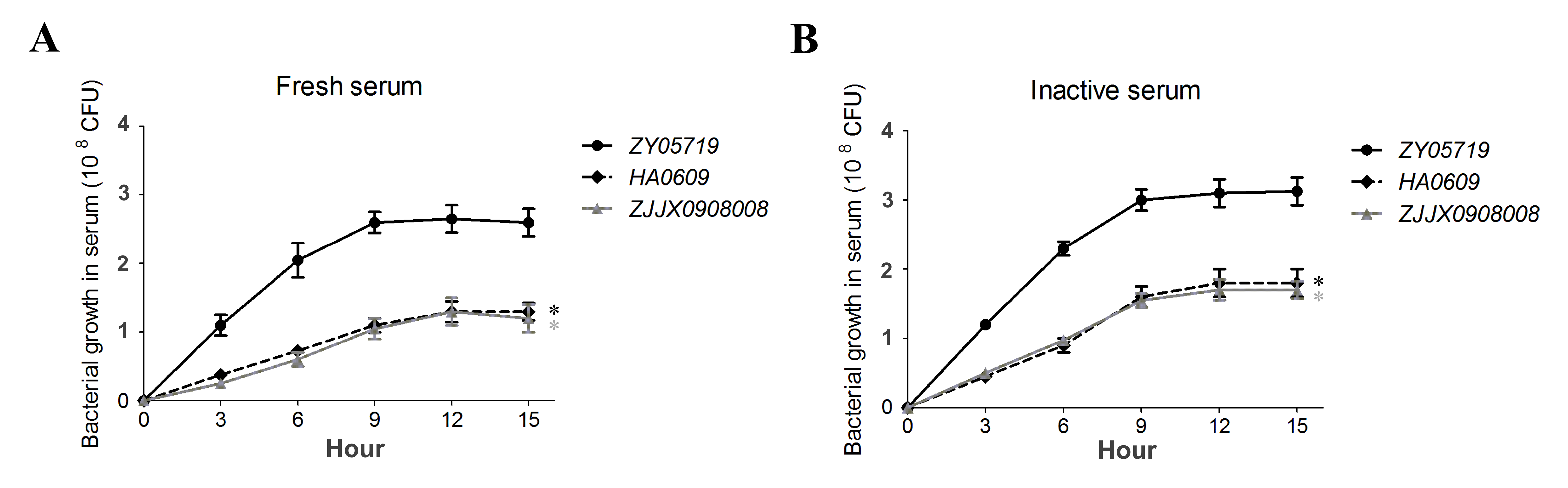

Supplement: Supplementary file 1 [file pathogens-09-00387-s001.zip › Figures/Fig. 1.tif]
